# Clinical Neuropathology teaching case 4-2015: Heterogenous brain pathologies temporally and spatially coinciding in limbic regions 

**DOI:** 10.5414/NP300884

**Published:** 2015-05-22

**Authors:** Gabor G. Kovacs, Johannes A. Hainfellner

**Affiliations:** Institute of Neurology Medical University of Vienna, Vienna, Austria

**Keywords:** argyrophilic grain disease, limbic encephalitis, prion disease, tauopathy

## Abstract

No Abstract available.

## Background 

Co-occurrence of neurodegenerative proteinopathies is increasingly recognized to be a frequent event in the brains of symptomatic and asymptomatic elderly individuals [1]. Prion diseases are rapidly progressive neurodegenerative conditions, which may pose differential diagnostic challenges. Co-occurence of diverse pathologies in prion disease is rarely reported, although occasionally unusual combinations may be detected [2]. We present here a case in which hetereogenous brain pathologies temporally and spatially coincided in medial temporal regions. 

## Case 

**Figure 1.**
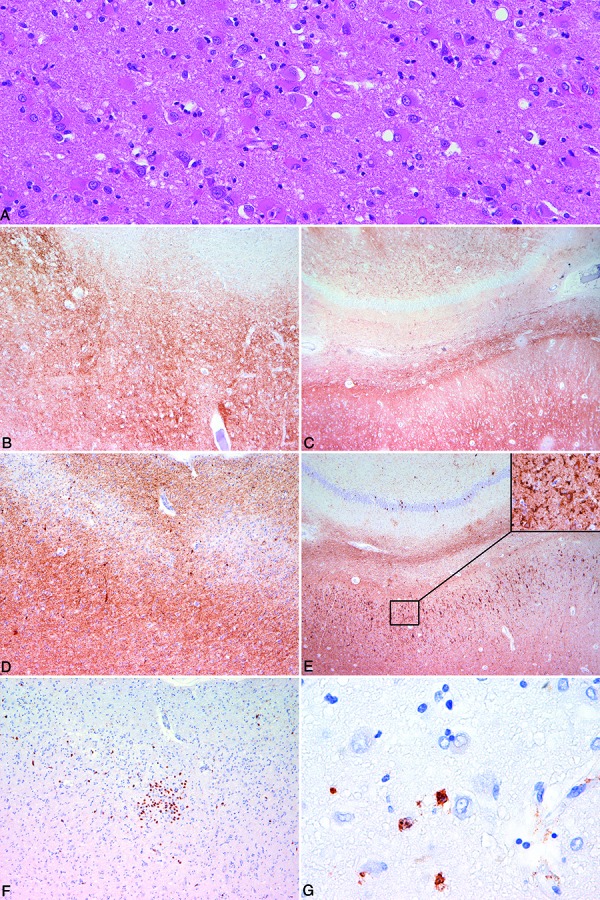
Figure 1.

A 72-year-old man who had progressive neurological symptoms, including dementia and disorientation, died after status epilepticus. Diagnostic neuropathological examination was performed and blocks of neocortical areas, basal ganglia, thalamus, brainstem, cerebellum, amygdala, and hippocampal formation were sampled. Severe loss of neurons was noted in the limbic system associated with reactive astrogliosis but with only mild degree of spongiform change (Figure 1A). Vacuolation of the neuropil, as characteristic for prion disease, was observed in neocortical areas, basal ganglia, thalamus and cerebellar cortex. This was associated with diffuse/synaptic type of PrP immunoreactivity, which was unusually prominent in the hippocampus (Figures 1B, C). In addition, a limbic predominant 4R tauopathy, compatible with argyrophilic grain disease, was also observed. τ-pathology was characterized by grains in dendrites of neurons. In addition, many neurons with cytoplasmic staining (pretangles) were observed in the dentate gyrus, pyramidal neurons of the cornu ammonis, amygdala, and accumbens nucleus (Figures 1D, E). This was associated with bushy astrocytes in the amygdala and oligodendroglial coiled bodies in the white matter of the hippocampus and amygdala. These two neurodegenerative conditions were accompanied by inflammatory cell infiltrates (Figure 1F) consisting mostly of CD8-positive cytotoxic T-cells, including attacking of neurons (Figure 1G). Immunostaining for viral antigens including Herpes simplex virus was negative. In summary, some regions in the medial temporal lobe, including the entorhinal cortex (Figures 1B, D, F) showed simultaneous presence of etiologically heterogenous disorders: prion disease, tauopathy, and limbic encephalitis. 

## Link between neurodegenerative and inflammatory brain pathologies 

Over the past decade, novel forms of encephalitis associated with antibodies to cell-surface or synaptic proteins have been described [3]. Recent studies indicate that < 5% patients with sporadic Creutzfeldt-Jakob disease develop serum antibodies to some neuronal antigens [4]. Prion diseases may associate with various τ-pathologies including also primary tauopathies [5]. 

## Conclusion 

The present case further demonstrates the considerable variability of pathological alterations in the human brain, which may also coincide spatially and temporally, emphasizing the role of neuropathology to understand the biological complexity in the background of progressive clinical symptoms. 

## Conflict of interest 

The authors report no conflict of interest.
